# Chronic Obstructive Pulmonary Disease Mortality in Diesel-Exposed Railroad Workers

**DOI:** 10.1289/ehp.8743

**Published:** 2006-03-30

**Authors:** Jaime E. Hart, Francine Laden, Marc B. Schenker, Eric Garshick

**Affiliations:** 1 Channing Laboratory, Brigham and Women’s Hospital and Harvard Medical School, Boston, Massachusetts, USA; 2 Exposure, Epidemiology, and Risk Program, Department of Environmental Health and; 3 Department of Epidemiology, Harvard School of Public Health, Boston, Massachusetts, USA; 4 Department of Public Health Sciences, University of California–Davis, Davis, California, USA; 5 Pulmonary and Critical Care Medicine Section, VA Boston Healthcare System, Boston, MA, USA

**Keywords:** chronic obstructive pulmonary disease, COPD, nonmalignant respiratory disease, occupational exposure, vehicle emissions

## Abstract

Diesel exhaust is a mixture of combustion gases and ultrafine particles coated with organic compounds. There is concern whether exposure can result in or worsen obstructive airway diseases, but there is only limited information to assess this risk. U.S. railroad workers have been exposed to diesel exhaust since diesel locomotives were introduced after World War II, and by 1959, 95% of the locomotives were diesel. We conducted a case–control study of railroad worker deaths between 1981 and 1982 using U.S. Railroad Retirement Board job records and next-of-kin smoking, residential, and vitamin use histories. There were 536 cases with chronic obstructive pulmonary disease (COPD) and 1,525 controls with causes of death not related to diesel exhaust or fine particle exposure. After adjustment for age, race, smoking, U.S. Census region of death, vitamin use, and total years off work, engineers and conductors with diesel-exhaust exposure from operating trains had an increased risk of COPD mortality. The odds of COPD mortality increased with years of work in these jobs, and those who had worked ≥ 16 years as an engineer or conductor after 1959 had an odds ratio of 1.61 (95% confidence interval, 1.12–2.30). These results suggest that diesel-exhaust exposure contributed to COPD mortality in these workers. Further study is needed to assess whether this risk is observed after exposure to exhaust from later-generation diesel engines with modern emission controls.

Diesel exhaust is a complex mixture of particles (< 1.0 μm in diameter) and combustion gases. These particles have organic compounds adsorbed on an elemental carbon core that may be inhaled deep into the lung. Regulation of diesel-exhaust exposure in the United States has been largely based on its potential to be a human lung carcinogen [U.S. Environmental Protection Agency (EPA) 2002]). Information regarding the occurrence of nonmalignant respiratory effects in humans was determined to be inadequate (U.S. EPA 2002). Diesel exhaust is a common exposure because many occupational groups (underground miners; bridge and tunnel workers; dockworkers; truck drivers; farm-workers; auto, truck, and bus maintenance garage workers; operators of heavy construction equipment; and railroad workers) are regularly exposed to diesel exhaust at work [National Institute for Occupational Safety and Health (NIOSH) 1988]. Although occupational exposures to dusts and fumes have been shown to contribute greatly to the burden of chronic obstructive pulmonary disease (COPD) (Balmes et al. 2003; Christiani 2005; Hnizdo et al. 2002; Meldrum et al. 2005; Trupin et al. 2003), previous studies have had limited ability to examine the health effects of a specific occupational group or exposure.

Workers within the U.S. railroad industry have been exposed to diesel exhaust since the industry converted from steam to diesel locomotives after World War II (U.S. Department of Labor Bureau of Labor Statistics 1972). There have been few epidemiologic studies assessing whether exposure to diesel exhaust is associated with increased mortality due to non-malignant respiratory diseases (Bergdahl et al. 2004; Ulvestad et al. 2000). In this case–control study, we investigated a possible association between exposure to diesel exhaust from operating locomotives and COPD mortality.

## Materials and Methods

### Study population

The data set of railroad workers obtained with the assistance of the U.S. Railroad Retirement Board (RRB) has been described elsewhere (Garshick et al. 1987a; Larkin et al. 2000). Briefly, the RRB manages the retirement benefits for all U.S. railroad workers with ≥ 10 years of railroad employment. Next of kin can draw benefits only by notifying the RRB of the worker’s death; therefore, it was possible to conduct a case–control study of deaths in the railroad industry that included workers with long-term railroad employment.

### COPD case series

Between 1 March 1981 and 28 February 1982, with the cooperation of the RRB, we collected incident deaths of U.S. railroad workers. Workers were eligible for inclusion in the study if they were born in 1900 or later and had no mention of suicide, accidental causes, or unknown causes on the death certificate. The original case–control study was designed primarily to study the association of exposure to diesel exhaust from locomotives with lung cancer mortality. Results from the lung cancer mortality case–control study have been published previously (Garshick et al. 1987a). During death certificate coding, because a specific code for COPD was not available in the *International Classification of Diseases*, Eighth Revision [ICD-8; World Health Organization (WHO) 1967], we noted deaths where COPD was specifically listed as the underlying cause of death. These deaths, plus ICD-8 codes 490–493 (bronchitis, including chronic bronchitis, emphysema, and asthma), were considered to be cases for this analysis. There were 536 cases with COPD or these related conditions listed as the underlying cause.

### Control series

Controls were selected from the pool of remaining deaths. This pool included cancer deaths other than lung cancer and deaths originally selected to be controls in the lung cancer study. Persons were excluded from the control group if they had lung cancer (ICD-8 code 162) listed anywhere on the death certificate, or other causes potentially related to exposure to diesel exhaust or fine particulate matter (PM). Because there is a potential association between diesel exhaust or fine particle exposure with selected cardiovascular diseases (deaths with ICD-8 codes 410–414, 420–425, or 427–429) or with bladder cancer (ICD-8 code 188) (Boffetta and Silverman 2001), individuals with these causes listed anywhere on the death certificate were not included in the control group. The selection of these cardiovascular ICD-8 codes was based on associations between cardiovascular mortality and fine PM reported by Pope et al. (2004). To assess the sensitivity of the results to the selection of the control group, we also conducted analyses excluding all cardiovascular deaths (ICD-8 codes 390–458) as controls. Deaths with COPD or related conditions listed as a secondary cause of death or other chronic respiratory diseases (ICD-8 codes 517–518) as primary or secondary cause were also not included as controls. After these exclusions, 1,525 individuals remained to serve as controls. The protocol was approved by the Brigham and Women’s Hospital and VA Boston Healthcare System institutional review boards.

### Exposure to diesel exhaust

Diesel exposure was determined based on yearly job code provided by the RRB. Since 1959, the RRB has maintained a computerized work history for all workers including a yearly Interstate Commerce Commission job code and number of months worked during the year. Transition from steam to diesel locomotives began largely after World War II. In 1946, 10% of the locomotives in service were diesel powered, and by 1959, 95% of locomotives in use were diesel powered (U.S. Department of Labor Bureau of Labor Statistics 1972). Therefore, we chose 1959 as the effective start of diesel exposure for our primary analyses. Alternatively, we conducted analyses using 1946 as the start of exposure to account for work during the transition period. Because it is unusual for railroad workers to change jobs and because yearly job codes were unavailable before 1959, job in 1959 was used to indicate pre-1959 exposure category. Using information from an industrial hygiene survey that we conducted (Woskie et al. 1988a, 1988b) and review of industry practices, subjects were characterized as exposed or unexposed to diesel exhaust based on yearly job code. The engineers (engineers and firemen) and conductors (conductors, brakemen, and hostlers) who worked on operating trains were determined to be “diesel exposed.” Other jobs, including ticket agents, station agents, signal maintainers and other maintenance of way workers, car repair workers, and clerks, were considered “unexposed.” We determined that the shop job codes were not specific for locomotive shops and that the shop workers should be considered a separate group containing a mixture of diesel-exposed and unexposed workers. From the industrial hygiene survey, information was available on mean level of cigarette-smoke–adjusted respirable PM for each of the major job groups. Mean levels for workers on operating trains, that is, engineers and conductors, were 71 μg/m^3^ and 89 μg/m^3^, respectively. Mean levels were lower for workers with clerical jobs (33 μg/m^3^) and signal maintainers (58 μg/m^3^). Because of uncertainties in assigning individual-level historical exposures, analyses were conducted assessing COPD risk between exposed and unexposed workers rather than specifically incorporating the PM exposure estimates. Diesel-exhaust exposure was defined by cumulative years of work in the engineer or conductor job group starting in 1959. Shop exposure was defined by cumulative years of work in the shop job group starting in 1959.

### Possible confounders

Information was available on several potential confounders. Given the small number of minorities in the database, individuals were coded as Caucasian or other race. Using the state of death from the death certificate, U.S. Census region of death was assigned as Northeast, South, Midwest, or West (Bureau of the Census 1993), to control for geographic variation. Questionnaires completed by next of kin provided information on smoking history; ever use of vitamins C, A, and E and multivitamins (which may protect against the development of COPD) (Anto et al. 2001; MacNee 2000; Romieu and Trenga 2001; Viegi et al. 2001); and the population (1–2,499, 2,500–49,999, ≥ 50,000 persons, or unknown) of the place where the deceased had lived for most of his life as an indicator of urban or rural location. Smoking status was coded as “never smoker” if the deceased never smoked, as “current smoker at death” if the deceased had smoked within the year of death, as “former smoker” if the smoker had stopped smoking before the year of death, and as “unknown smoker” if smoking information was not available. Average number of cigarettes smoked and age started and stopped smoking were used to calculate pack-years of cigarettes, and years quit smoking was calculated for all former smokers by subtracting age last smoked from age at death. Indicator variables were created for missing information. Total years of retirement were calculated by subtracting year of retirement from year of death and were used to account for a potential healthy worker survivor effect, an effect where workers who remain in the workplace tend to be healthier than those who leave (Arrighi and Hertz-Picciotto 1993, 1994). Occupational categories with the potential for asbestos exposure were available from the original case–control study (Garshick et al. 1987a). Classification was based on the results of a survey of railroad employees (Garshick et al. 1987b) and on a review of railroad, medical, and industrial literature and was included as an indicator variable. Other exposures associated with railroad work included silica as a result of track sanding operations (Woskie et al. 1988a). However, specific information regarding this potential exposure was not available to us.

### Statistical analysis

We estimated the association between diesel-exhaust exposure and COPD mortality using logistic regression and present odds ratios (OR) and 95% confidence intervals (CIs). When appropriate, tests for trend were performed using ordinal variables (0, 1, 2, 3) for increasing exposure categories. All analyses were performed in SAS (version 8; SAS Institute Inc., Cary, NC).

## Results

Characteristics of the cases and controls are presented in Table 1. Fifty-nine percent of the controls died from malignant neoplasms, and 23% from nonexcluded cardiovascular causes. Cases were more likely to have been smokers, to smoke more cigarettes on average, to be Caucasian, and to have died outside of the Northeast.

Logistic regression results are presented in Table 2, categorizing diesel exposure based on years of work in the engineer or conductor job group starting in 1959, and shop exposure as years of work in a shop job starting in 1959. In the unadjusted model, there was no consistent association between work in a diesel-exposed job and COPD mortality. There was also no consistent association with work in a shop job. After adjustment for age, there was a trend apparent only in the diesel-exposed group. After adjustment for age at death, race, and the healthy worker survivor effect, the highest ORs were for workers with ≥ 16 years of work in both the diesel-exposed and shop categories. However, the *p*-value for trend was again significant only for the diesel-exposed group. Adjustment for smoking (smoking status, pack-years, and years quit) attenuated the ORs in the diesel-exposed group, but the *p*-value for trend remained significant. The smoking-adjusted OR for each additional year of work as an engineer/conductor was 1.02 (95% CI, 1.01–1.04). After smoking adjustment, there was less evidence of a relationship between years of work and COPD mortality in the shop workers, but risks were generally elevated. Additional models using smoking duration and average amount smoked per day to adjust for smoking gave similar results. Adjustment for other possible confounders (Census region of death, asbestos exposure, and vitamin C, A, and E and multivitamin use) did not substantially change the risk estimates and were not significant predictors of COPD mortality. Similar analyses were conducted based on exposure starting in 1946. The ORs adjusted for age, race, smoking, and healthy worker survivor effect for years of work after 1946 in a diesel-exposed job were 0–20 years, OR = 0.60 (95% CI, 0.36–1.01); 21–25 years, OR = 1.08 (95% CI, 0.75–1.56); 26–30 years, OR = 1.58 (95% CI, 1.12–2.22); and ≥ 31 years, OR = 1.82 (95% CI, 1.13–2.94). For the shop worker group, the ORs for years of work after 1946 ranged from 1.17 in the group with 0–20 years of work after 1946 to 2.21 for those with ≥ 30 years; no consistent pattern of increasing risk with increasing years of work was observed. In analyses where deaths from all cardiovascular causes were excluded from the control series, similar results were found (data not shown).

## Discussion

In this study of diesel-exposed railroad workers, after adjustment for active smoking and other covariates, work in diesel-exposed jobs was associated with higher risks of COPD mortality compared with work in unexposed jobs. These risks increased with increasing years of work. The greatest risks were observed for those individuals with the longest work on operating trains (multivariate-adjusted OR = 1.61; 95% CI, 1.12–2.30) for engineers/conductors with ≥ 16 years of work starting in 1959 (*p*-value for trend = 0.02). The shop worker group, which likely includes a mixture of both diesel-exposed and unexposed workers, did not demonstrate a significant trend with increasing years of work after 1959, although the ORs were elevated. In our retrospective cohort study of 54,973 railroad workers, we also observed a similarly elevated relative risk (RR) of 1.41 (95% CI, 1.27–1.55) for COPD mortality between 1959 and 1996, comparing workers in the engineer and conductor group in 1959 with workers in the unexposed group (Garshick et al. 2004). In contrast to our current case–control study, however, we were not able to directly adjust for smoking because this information was not available.

The U.S. railroad industry converted from steam to diesel-powered locomotives after World War II, with a rapid increase through the 1950s. First-generation diesel locomotives introduced during the 1940s and 1950s were described as “smokier” than locomotives introduced later, although historical exposure measurements are not available (Eschenroeder 2004; Woskie et al. 1988a, 1988b). In addition, some railroads operated these locomotives with the cab in the rear, a configuration that increased exposure because the exhaust stack was located in front of the cab rather than behind it (Liukonen et al. 2002; Verma et al. 2003). Locomotives introduced in the 1960s and later in the 1980s had improved emission characteristics. Our research group conducted an exposure assessment in the early 1980s in four smaller railroads that used a combination of first- and second-generation locomotives. No specific marker of diesel exposure was measured, but workers on operating trains had mean respirable PM levels adjusted for secondhand smoke approximately two to three times that of unexposed clerical workers (71 μg/m^3^ and 89 μg/m^3^ of cigarette-adjusted PM vs. 33 μg/m^3^) (Woskie et al. 1988a, 1988b). These results indicate that railroad workers who were on operating trains and whose COPD mortality was assessed in this study were more exposed to diesel exhaust than workers not on operating trains, and most likely had greater exposures than contemporary workers.

In the present study, we considered diesel exposure to start in 1959, the date that the railroad industry had largely converted from steam to diesel-powered locomotives. However, the overall proportion of diesel locomotives in service in large railroads was 27% in 1949 and 55% in 1952 (U.S. Department of Labor Bureau of Labor Statistics 1972). Therefore, many of the workers in the study probably had between 5 and 10 years or more of additional exposure to diesel exhaust before 1959, possibly influencing the relationship between years of exposure and COPD mortality. We attempted to account for work before 1959 by assessing exposure starting in 1946. As in the analysis based on exposure starting in 1959, workers with the greatest duration of work in jobs with diesel exposure had the greater risk of COPD mortality. It was not possible to account for historical differences in emission characteristics of locomotives and work practices when calculating years of exposure. However, misclassification of exposure would be nondifferential and thus would bias the results toward the null. Another possible source of exposure misclassification is from PM attributable to steam combustion products before the transition to diesel, and it is possible that this also contributed to mortality. Although there is no study of the PM size distribution from steam locomotives, studies of the PM emitted from coal-fired boilers indicate that only a small percentage (4–6%) are in the fine range and respirable, that is, < 2.5 μm in diameter (Chang et al. 2004). This suggests that diesel combustion PM is more likely to be inhaled deeply into the lung and to more strongly contribute to the effects of exposure on COPD mortality.

The shop worker group also included workers with diesel exposure, but also with exposure to other dusts and fumes from locomotive and nonlocomotive repair shop operations. After adjustment for cigarette smoking, there was no relationship between years of work and COPD mortality, although workers with the longest duration of work did have the highest risk. It is possible that exposure to various dusts and fumes generated in railroad shops before 1959 in addition to diesel exposure influenced COPD mortality. It is also possible that the mix of shop workers in diesel locomotive shops and in shops not responsible for locomotive repair who were not exposed to operating trains contributed to the lack of a dose response in that group, because there was no way to separate workers with and without exposure using job titles. The results for the shop workers may also be imprecise because of few subjects in the two top cumulative exposure categories.

Our results are consistent with previous studies relating occupational exposures to dusts and fumes to the development of COPD, and air pollution studies where exposure to PM is associated with both hospitalizations and COPD mortality (Chew et al. 1999; Harre et al. 1997; Sunyer 2001). Occupational exposures to mineral dust, welding and metal fumes, inorganic and organic dusts, and vehicle exhausts have been implicated as potentially important risk factors for COPD, including in nonsmoking individuals (Anto et al. 2001; Balmes et al. 2003; Becklake 1989; Christiani 2005; Coggon and Newman Taylor 1998; Garshick et al. 1996; Korn et al. 1987; Mastrangelo et al. 2003; Meldrum et al. 2005; Oxman et al. 1993; Trupin et al. 2003; Ulvestad et al. 2000; Viegi and Di Pede 2002; Viegi et al. 2001). Additionally, in the Third National Health and Nutrition Examination Study, 19.2% of all U.S. COPD cases and 31.1% of nonsmoking cases were attributed to work exposures (Hnizdo et al. 2002).

Experimentally, exposure to the organic compounds found in diesel exhaust and on the surface of the particle have also been linked to allergy, airway inflammation, and changes in airway function (Prieto et al. 2000; Riedl and Diaz-Sanchez 2005; Rudell et al. 1996; Saxon and Diaz-Sanchez 2000). Air toxics and other polycyclic aromatic hydrocarbon compounds found in diesel exhaust may be important in the induction of such airway inflammatory changes and possibly oxidative stress in the lung (Ma and Ma 2002). Taken together, these studies support the hypothesis that occupational exposure to diesel exhaust can contribute to the occurrence of COPD and COPD mortality even after exposure has ceased.

Since the 1950s, researchers have shown that acute air pollution exacerbates existing COPD and asthma and also increases their incidence. This research includes panel studies and time series studies of daily variation in hospitalizations (Pope 2000; Schwartz et al. 1996). Furthermore, studies examining the long-term effects of air pollution exposure have consistently found increased prevalence of symptoms or diagnoses of emphysema or COPD for areas with higher levels of PM (Abbey et al. 1995; Schwartz 1993; Schwartz et al. 1996; Sunyer 2001). In residents living in areas of high air pollution, small airway fibrosis and PM deposition were noted in small airways, suggesting that chronic exposure to PM results in pathologic changes in the lung (Brauer et al. 2001; Dai et al. 2003). Similar findings have been noted in workers with occupational dust exposures (Churg and Wright 1983, 1985). Therefore, our findings of an association of COPD mortality with occupational exposure to diesel exhaust are consistent with observations from the occupational health and air pollution literature.

There are several possible sources of limitation in this study, including classification of outcome from death certificates, classification of exposure based on job title and an industrial hygiene survey, and information on confounders from next of kin. Death certificates have been shown to underestimate the true number of workers with severe COPD at death. In the Tucson Epidemiologic Study of Obstructive Airways Disease (Camilli et al. 1991), 25% of deaths with clinically documented moderate to severe obstructive lung disease were identified using underlying cause of death only, whereas 81% had COPD noted as either underlying or contributing cause on the death certificate. Similarly for asthma, in a cohort of persons from Olmstead County, Minnesota (which excluded persons with COPD based on detailed laboratory and clinical criteria), the specificity was 99% but the sensitivity was only 42% for detecting asthma based on death certificate diagnosis (Hunt et al. 1993). Because it would be unlikely for a physician to report obstructive lung disease based on diesel exposure category, misclassification would be nondifferential and the observed ORs are likely attenuated. In models considering COPD from both underlying and contributing causes, similar results were observed to those using only underlying cause.

In any COPD study, cigarette smoking is an important potential confounder of the exposure–disease association. Information on smoking status was available only from next of kin. Proxy respondents have been shown to accurately report cigarette smoking status, including duration of smoking and amount smoked (Kolonel et al. 1977; Rogot and Reid 1975). There were, in fact, small differences in smoking rates by job title, with a slightly higher rate of smoking among the diesel-exposed workers (Larkin et al. 2000). We were able to account for these differences in the smoking-adjusted analyses, and it is unlikely that residual confounding explains the current associations.

## Conclusion

In this case–control study of railroad workers, work in jobs with exposure to diesel exhaust was associated with increased mortality from COPD. These elevations persist after controlling for smoking and increased with increasing years of work in exposed jobs. Further study of incident cases in populations exposed to diesel exhaust are needed to assess the robustness of this relationship and whether the relationship is observed after exposure to exhaust from later-generation diesel engines and with modern emission controls.

## Figures and Tables

**Table 1:** Basic characteristics of COPD cases (*n* = 536) and controls (*n* = 1,525).

	Cases	Controls
Characteristic	No. (%)	No. (%)
Age at death (mean ± SD)	536 (72.3 ± 7.0)	1,525 (70.0 ± 8.1)
Total years of work (mean ± SD)	536 (28.4 ± 6.1)	1,525 (29.4 ± 6.3)
Years off work (mean ± SD)	536 (10.5 ± 5.2)	1,525 (8.6 ± 5.4)
Pack-years of cigarettes (mean ± SD)^a^	424 (58.0 ± 37.4)	961 (48.0 ± 39.4)
Years quit smoking (mean ± SD)^b^	268 (11.6 ± 9.9)	555 (15.9 ± 12.9)
Job title in 1959		
Engineer	75 (14.0)	154 (10.1)
Conductor	104 (19.4)	292 (19.2)
Shop worker	85 (15.9)	232 (15.2)
Unexposed	272 (50.7)	847 (55.5)
Smoking status		
Never	23 (4.3)	266 (17.4)
Current	156 (29.1)	406 (26.6)
Former	268 (50.0)	555 (36.4)
Unknown	89 (16.6)	298 (19.5)
Population of usual place of residence		
≥50,000	204 (38.1)	538 (35.3)
2,500–49,999	168 (31.3)	506 (33.2)
1–2,499	69 (12.9)	171 (11.2)
Unknown	95 (17.7)	310 (20.3)
Region at death		
Northeast	81 (15.1)	310 (20.3)
West	113 (21.0)	260 (17.1)
Midwest	174 (32.5)	510 (33.4)
South	168 (31.3)	445 (29.2)
Caucasian race	499 (93.1)	1,321 (86.6)
Vitamin use		
Vitamin A	23 (4.3)	78 (5.1)
Vitamin C	65 (12.1)	231 (15.2)
Vitamin E	59 (11.0)	167 (11.0)
Multivitamin	167 (31.2)	417 (27.3)

Values shown are number (%) except where indicated.

a Pack-years of cigarettes calculated for current and former smokers.

b Years quit smoking calculated for former smokers.

**Table 2:** OR (95% CI) of COPD mortality based on cumulative years of work by job group compared with unexposed workers.

	Engineer/conductor (years of work)				Shop workers (years of work)			
Job group	> 0–10	11–15	≥ 16	*p*-Value for trend^a^	> 0–10	11–15	≥ 16	*p*-Value for trend^a^
Cases (*n*)	48	59	75		60	30	16	
Controls (*n*)	138	133	221		157	85	64	
Regression model								
Unadjusted	1.01 (0.71–1.45)	1.30 (0.93–1.82)	0.99 (0.74–1.34)	0.73	1.12 (0.81–1.55)	1.03 (0.67–1.61)	0.73 (0.42–1.29)	0.55
Age adjusted	0.94 (0.63–1.30)	1.29 (0.92–1.81)	1.44 (1.04–1.99)	0.02	1.10 (0.79–1.53)	1.02 (0.65–1.59)	1.02 (0.57–1.81)	0.73
Age, race, HWSE adjusted^b^	0.82 (0.56–1.18)	1.41 (1.00–2.00)	1.83 (1.29–2.60)	0.001	1.08 (0.77–1.51)	1.22 (0.78–1.91)	1.37 (0.75–2.49)	0.17
Age, race, HWSE, smoking adjusted^c^	0.75 (0.51–1.09)	1.35 (0.94–1.93)	1.67 (1.17–2.39)	0.01	1.29 (0.90–1.84)	1.25 (0.79–2.00)	1.36 (0.74–2.52)	0.09
Multivariable adjusted^d^	0.75 (0.51–1.10)	1.33 (0.93–1.91)	1.61 (1.12–2.30)	0.02	1.32 (0.92–1.90)	1.25 (0.78–2.01)	1.37 (0.74–2.55)	0.08

HWSE, healthy worker survivor effect.

a Test for trend performed using ordinal categories (1, 2, 3) for the increasing years of exposure categories.

b Adjusted for the HWSE using total years off work.

c Adjusted for age, race, HWSE, smoking status (current, former, never), pack-years, and years since quit smoking.

d Adjusted for age, race, HWSE, smoking status (current, former, never), pack-years, years since quit smoking, U.S. Census region of death, vitamin C use, and years off work.
